# Spanish Version of the Measure of Processes of Care (20 Items): Psychometric Properties

**DOI:** 10.3390/children12070871

**Published:** 2025-07-01

**Authors:** Manuel Pacheco-Molero, Catalina Patricia Morales-Murillo, Irene León-Estrada, Roberto Hernández-Soto, Mónica Gutiérrez-Ortega

**Affiliations:** 1 Faculty of Education, Universidad Internacional de La Rioja, 26006 Logroño, Spain; manuel.pacheco@unir.net (M.P.-M.); irene.leon@unir.net (I.L.-E.); 2 Faculty of Education, Universidad Católica de Valencia, 46001 Valencia, Spain; cp.morales@ucv.es; 3 Faculty of Education, Universidad Antonio de Nebrija, 28248 Madrid, Spain; rhernans@nebrija.es; 4 Faculty of Education and Social Work, Department of Pedagogy, University of Valladolid, 47005 Valladolid, Spain

**Keywords:** family-centered care, measurement of processes of care, service providers, psychometric assessment, MPOC-20, quality of services, early intervention

## Abstract

Background/Objectives: Family perceptions of family-centered services are important for improving processes and outcomes of services for children with disabilities or developmental risk. The Measure of Processes of Care 20-item version (MPOC-20) assesses family-centered practice from parents’ perspectives. This study examined for the first time the psychometric properties of the first Spanish version of the MPOC-20 in children with disabilities aged 0–18 years. Methods: A total of 659 families from 51 care services across Spain completed the MPOC-20, with participants randomly divided into two samples: one for exploratory factor analysis (EFA) and the other for confirmatory factor analysis (CFA). Results: The results confirmed a two-factor model, with the best fit for the dimensions of providing comprehensive and supportive care and providing information. Internal consistency analysis indicated strong reliability of the factor scores. Conclusions: The Spanish version of the MPOC-20 demonstrated good psychometric properties and is recommended for assessing the quality of family-centered services.

## 1. Introduction

Family-centered services (FCS) are considered the best option in service delivery for children and their families [1]. FCSs are characterized by a comprehensive approach to the family and actively involving the family in the development of the intervention program [2]. As well as addressing the priorities of both the child and the family [3], it is recognized that the members of each family are unique and the experts on the child’s skills and needs [4]. FCS are associated with positive outcomes for children and their families [5].

Implementation of FCSs requires periodic evaluations to detect how care is being delivered by practitioners [6]. FCS professionals have a responsibility to establish a cooperative relationship with families and their children [7]. Family perceptions of FCSs are important for improving processes and outcomes of services for children with disabilities or developmental risk [8].

The Measure of Processes of Care (MPOC) is an instrument developed by the CanChild Centre for Childhood Disability Research and it is currently one of the most widely used tools for assessing FCS and perceptions of the care process [9,10]. Specifically, the MPOC-20 determines the extent to which the service provided to its clients is family-centered attention [11]. This scale has been shown to be reliable and valid, supported by numerous research studies in diverse populations and clinical settings, leading to its widespread adaptation in different countries [12]. Hence, the use of the MPOC-20 across different clinical and cultural contexts supports its comparability and applicability in cross-national investigations, being an advantage over the use of similar instruments focused on families’ perceptions such as the Family-Centered Practices Scale (FCPS), the Parent Satisfaction with Family-Centered Practices (PSFCP) or the Family-Centered Care Self-Assessment Tool (FCC-SAT).

In order to establish a collaboration between families and professionals, FCS must be perfectly defined, to align the perceptions of both [13]. Therefore, the importance of adapting and validating MPOC in different cultural and linguistic contexts is highlighted [12]. In Spain, families with children with developmental challenges received services of different characteristics (health, education, and social services). Measures are needed to determine if the services provided are family-centered, under family’s perceptions.

The Spanish version of the MPOC might allow evaluation of care services for children with disabilities in that context. Hence, it would be necessary to test its psychometric properties.

Therefore, this study has the following objectives: (i) To explore the factorial distribution (EFA) of the items of the MPOC-20 according to data collected in a sample of families (Sample 1) with children under 18 receiving services in Spain. (ii) To confirm the factor structure (CFA) of the MPOC-20 derived from the EFA in data collected in a second sample of families (Sample 2) with children under 18 receiving services in Spain. (iii) To analyze the internal consistency (reliability) of the MPOC-20 factor scores in two samples of Spanish families (Sample 1 and Sample 2) with children under 18 receiving services in Spain.

## 2. Materials and Methods

### 2.1. Translation Process

CanChild is a research and educational center that provides evidence-based information to improve the lives of children and youth with disabilities and their families. To adapt the MPOC-20 for the Spanish context, written permission was obtained from CanChild, which holds the publication rights to the original scale. Based on established guidelines from prior studies on translation and cross-cultural adaptation, advanced and iterative translation methods were employed. In the first phase, four independent professionals with at least 10 years of experience in early childhood care each translated the MPOC-20 into Spanish. These translated versions were then consolidated into an initial joint version (Phase 2). This preliminary version was subsequently back-translated into English by two professional translators (Phase 3), allowing for the identification of potential discrepancies and ensuring semantic fidelity to the original instrument. The final version of the translation was then reviewed and approved by CanChild.

The Spanish pre-version of the MPOC-20 was assessed by an expert panel consisting of child disability researchers and clinicians (Phase 4). This panel evaluated the adaptation for appropriateness and comprehensibility, concluding that no further cultural adaptations were needed, and approved the translation. Finally (Phase 5), the completed versions were sent to CanChild for formal approval. Feedback confirmed that the questions were clear and suitable for parents in Spain.

### 2.2. Participants

Participant families (N = 659) were recruited from 51 care services throughout Spain. The Ethics Review Committee at Universidad Internacional de La Rioja approved our study (approval: PI025/2022) on 11 July 2022. Respondents gave written consent for review and signature before starting the study. As further explained in the data analysis section, these families were randomly assigned to one of two sample groups (Sample 1 or Sample 2). Hence, sociodemographic data of the families is presented by sample groups (Table 1). The mean age in years of caregivers who completed the questionnaires was 37.87 (SD = 6.15, range 20 to 62 years old) for Sample 1 and 37.92 (SD = 6.05, range 21 to 70 years old) for Sample 2. The results did not show statistically significant differences between sample groups were identified in relation to the age of the caregiver completing the questionnaires. Most caregivers who completed the questionnaire were female (87.10%), the mother of children receiving services (87.10%), had completed undergraduate or graduate studies (40.36%), and reported having a median socioeconomic status -SES- (74.66%). Chi-squared results did not show statistically significant differences between the two samples in relation to these variables (Table 1).

The number of children receiving services per family ranged from 1 to 3 children, with 1 child per family (94.54%) being the most common answer among both sample groups (Table 2). Children were mostly male (74.51%), needed supervision (46.13%) or assistance to complete tasks (25.19%), and were between 1 and 3 years old (43.25%) and 3 and 5 years old (44.61%). The results did not show statistically significant differences were identified between sample groups.

Most families in both sample groups had been receiving services from 12 to 23 months (20.04%) by the time they completed the questionnaires, followed by less than 6 months (28.53%) and 6 to 12 months (22.76%). Families tended to receive services once (52.96%) or twice (23.97%) a week and were reported to interact with the service provider for about 95% of the session in 69% of the cases (Table 3). The results did not show statistically significant differences between sample groups were identified for these variables either.

### 2.3. Measures

The MPOC-20 is a self-administered instrument that exhibits internal consistency ranging from 0.83 to 0.90 (Cronbach’s alpha) and test–retest reliability between 0.78 and 0.86 (intraclass correlation coefficient—ICC) [14]. Responses vary from 1 to 7 in a Likert-type scale as follows: 7 (to a very great extent), 6 (to a great extent); 5 (to a fairly great extent); 4 (to a moderate extent); 3 (to a small extent); 2 (to a very small extent); 1 (not at all). The MPOC-20 are divided into five domains: enabling and partnership (EP), providing general information (PGI), providing specific information about the child (PSI), coordinated and comprehensive care for child and family (CCC), and respectful and supportive care (RSC). Scores for individual items are combined to calculate the ultimate score for each domain [8].

### 2.4. Data Analysis

Data was split in two samples [15]. Sample 1 was used for running an exploratory factor analysis (EFA), and Sample 2 was used to confirm the factor structure resulting from the EFA run with Sample 1 with a confirmatory factor analysis (CFA). For splitting the data at random [16], each participant was assigned a number between 1 and 659. A website (https://www.alazar.info/generador-de-numeros-aleatorios-sin-repeticion access date: 1 June 2025) was used to generate 329 nonrepetitive random numbers between 1 and 659. Participants who corresponded to those 329 generated random numbers were selected for Sample 1 (n = 329), the remaining participants, for whom no random number was matched, were included in Sample 2 (n = 330). The sample size of both samples was adequate to run the analysis, following the recommendations of 5 to 10 participants per number of variables provided [17]. The MPOC-20 consists of 20 items, therefore a minimum sample of 200 participants was required for both samples.

Chi-squared statistics were used to identified differences based on the sociodemographic characteristics of the participants among the two samples. Non-statistically significant differences (*p* > 0.05) among the two sample groups supported the equivalence of the samples for running the EFA with Sample 1 and then confirming the results (CFA) with Sample 2 (Table 1, Table 2 and Table 3). In addition, Cramer’s V effect sizes were calculated to assess the effect size of sample group over the sample sociodemographic variables. Values of Cramer’s V between 0.07 and 0.21 indicate a weak effect, values between 0.21 and 0.35 indicate a moderate effect, and values greater than 0.35 indicates a strong effect [18]. Effect sizes were less than weak in all the sociodemographic variables, indicating that being part of Sample 1 or Sample 2 did not had an effect on differences on the tested variables. Hence, the equivalence of Sample 1 and Sample 2 is supported for running the analyses.

JASP software [19] version 0.18.3 was used for running the EFA and CFA. To assess if the matrices had high levels of common variance, which were suitable for factor analysis, the Kaiser–Meyer–Olkin test was run [20,21]. KMO results above 0.50 were considered acceptable [22]. In addition, the Bartlett test was also used to test the adequacy of the data for running the analysis [23]. The Bartlett’s test of sphericity was used to determine if there was some overlap among variables (i.e., items), which then could be summarized in factors, and *p*-values < 0.01 indicated our samples’ data were suitable for grouping the items among factors.

The EFA extraction method was parallel analysis, the estimation method minimum residual, and the rotation method used was oblique with Promax. Only factor loadings with weights >0.40 were accepted [24,25]. Items with double loadings were assigned to one of the factors after considering the weight of the loading and the theoretical adequacy of the item depending on the concepts being measure by the factors. To ensure no multicollinearity among factors, a correlation analysis was run [26]; r-values < 0.80 indicated no multicollinearity among factors [27].

For CFA, the results did not show statistically significant chi-squared results (*p* > 0.05), and the comparative fit index (CFI) and goodness of fit index (GFI), a Bentler–Bonett normed fit index (NFI) above 0.95, and root mean squared error of approximation (RMSEA) and standardized root mean square residual (SRMR) below 0.06 and 0.08, respectively, were considered indicators of good fit of the data to the hypothesized model [28]. Finally, statistically significant factor loadings (*p* < 0.01) and weight values > 0.50 were considered indicators of convergent validity of the items [29]. Finally, the magnitude of factor loadings was interpreted following the three levels (λ = 0.30 (weak), 0.60 (medium), and 0.90 (strong)) [30].

## 3. Results

This study aimed to test the MPOC-20 items’ distribution among factors in two equivalent samples of families receiving services in Spain and to determine the internal consistency of the factor scores. Both EFA (Sample 1) and CFA (Sample 2) were run to explore the items distribution among factors (EFA) and then to confirm the identified factor structure (CFA). KMO and Bartlett’s test of sphericity results supported the adequacy of the sample datasets for running the analysis: the Sample 1 KMO = 0.96 and Sample 2 KMO = 0.94; Sample 1 Bartlett’s test results were χ^2^ = 7428.81, df = 190, *p* < 0.001, and Sample 2 Bartlett’s test results were χ^2^ = 4520.86, df = 190, *p* < 0.001.

Sample 1 EFA results supported a two-factor model; Factor 1 explained 34% of the variance and Factor 2 explained 30%, while both factors explained 64% of the variance. Both factors were positively correlated: r = 0.77, and because r < 0.80, no multicollinearity was identified. Factor loading weights are shown in Table 4. Only factor loadings above 0.40 were accepted, all items had at least one factor loading above 0.40.

Factor 1 consisted of 11 items (i.e., i3, i13, i10, i9, i5, i12, i8, i6, i1, i11, i4), these items were related to providing comprehensive and supportive care (e.g., i3 = Professionals provide a caring atmosphere rather than just give you information?). Factor 2 included 9 items (i.e., i7, i19, i20, i16, i17, i18, i14, i2, and i15). Factor 2 items were associated with providing information (e.g., i14 = Professionals provide you with written information about your child’s progress?).

Sample 2 CFA results confirmed the EFA identified the factor structure for the Spanish Sample 1. Chi-squared results were above 0.05, indicating there was no difference between the hypothesized model and the data fit: χ^2^ = 81.42, df = 169, *p* = 1.00. CFI (1.00), GFI (0.99), and NFI (0.99) indexes were above 0.95, indicating the model predicted between 99% and 100% of the variance. As for the errors, RMSEA = 0.00 and SRMR = 0.06, and these values indicated adequate error levels. All factor loadings were statistically significant, and β-values ranged from 0.66 to 0.87 (Table 5). When considering λ-values and the magnitude levels [30], eleven items were classified as having a strong magnitude (λ > 0.90, ranging from 0.91 to 1.34), eight as having a medium magnitude (0.90 > λ > 0.60, ranging from 0.66 to 0.81), and one as having a weak magnitude (0.60 > λ > 0.30, λ = 0.59).

Finally, internal consistency results support a strong reliability of the factor scores. Table 6 shows the reliability results by samples and factors.

## 4. Discussion

The translation and adaptation of the MPOC-20 Spanish version proved to be a reliable and valid instrument to assess the extent to which families perceived whether the services they received for their children were family-centered. The study highlights the distribution of the dimensions obtained in comparison with the original study [14] and the different validations carried out internationally. Originally, the items were distributed in five dimensions (EP, PGI, PSI, CCC, and RSC). The exploratory analysis showed that the best fit of the items was distributed in two factors: (1) providing comprehensive and supportive care, and (2) providing information. The confirmatory analysis confirmed the factorial structure, obtaining Cronbach’s alpha values between 0.92 and 0.94, which are optimal values reported in other validations. This difference in the distribution of the items has been reported by other authors who have translated and validated the MPOC-20 in other countries. In the South African validation, for example, the factor structure was also reorganized, and two dimensions—respectful and supportive care, and providing information and advice—were identified as the best fit. However, in that case, the scale was reduced to eight items [31].

In Brazil, low internal consistency and low reliability in the domains of enabling and partnership and coordinated and comprehensive care were reported [32]. These authors also warned about the services in Brazil and were cautious regarding the values obtained in the two previous dimensions. In test validations carried out in Singapore [33], Malaysia [34], and Jordan [35], the authors merged the dimensions from their factorial studies to four dimensions. The Turkish [36], Dutch [37], and Japanese [38] versions reported a factor distribution differing from that of the original study [14]. Therefore, the importance of performing a factor analysis to determine the validity and reliability of an instrument when in a culturally different environment is noted [39].

The internal consistency results of this study support a strong reliability of the tool. Comparing Cronbach’s alpha values of international validations, our study has among the highest thresholds reported [32,38,40].

Limitations of this study include that the MPOC-20 is a self-administered tool to measure parent’s perceptions about the services delivered and whether they are family-oriented. Hence, their subjective vision does not completely match with an objective one. Additionally, in the Spanish version of the MPOC-20 there was observed a reduction of the dimensions, from five to two. However, rather than implying a loss of information, this reduction may reflect underlying social and cultural factors that shape how parents interpret and report their perceptions of the services received. Another limitation is the lack of balance regarding sex in the sample, which may have influenced the responses. Finally, the current study did not explore the construct validity. Given that the Spanish version of the instrument is a cross-cultural adaptation, authors decided to explore other psychometric properties instead of construct validity, which is more associated with the development of a tool.

Future research could expand upon the results by exploring further validation (e.g., convergent and discriminant) and across Spanish-speaking countries to explore the model beyond the initial sample. Given the gender imbalance, future investigations could include more balanced samples and identify possible differences in perception of services. Additionally, cross-cultural comparison could be conducted between the Spanish version of the MPOC-20 and other validated versions (e.g., Turkish, Japanese, Brazilian) to analyze cultural variables among perceptions of family-centered care.

## 5. Conclusions

The Spanish version of the MPOC-20 demonstrated correct psychometric properties and can be recommended for evaluating family-centered services. This study has shown that the scale items best fit a two-factor structure, specifically the dimensions of providing comprehensive and supportive care and providing information. Hence, two dimensions should be prioritized when assessing the quality of services delivered to families. The MPOC-20 offers utility in both research and practical application, supporting the implementation of family-centered early intervention services. Its availability in Spanish enables the assessment of family-centered care across Spanish-speaking populations and contexts.

## Figures and Tables

**Table 1:** Observed frequency of sociodemographic variables related to participant families.

Variables	Sample 1	Sample 2	Total	*Χ* ^2^	*df*	*p*	Cramer’s *V*
Gender of caregiver				1.126	1	0.289	0.041
Female	282	292	574				
Male	47	38	85				
Total	329	330	659				
Relationship with the child				1.101	3	0.777	0.041
Mother	283	291	574				
Father	41	36	77				
Legal guardian	1	1	2				
Other	4	2	6				
Total	329	330	659				
Education of caregiver				4.293	2	0.117	0.081
Up to secondary	79	94	173				
Associates degree	122	98	220				
University/college degree	128	138	266				
Total	329	330	659				
Socioeconomic status (SES)				3.847	2	0.146	1.077
High	4	5	9				
Medium	256	236	492				
Low	69	89	158				
Total	327	324	659				

**Table 2:** Observed frequency of sociodemographic variables related to children receiving early intervention services.

Variables	Sample 1	Sample 2	Total	*Χ* ^2^	*df*	*p*	Cramer’s *V*
No. of children who receive services in the family				1.321	2	0.517	0.045
1 child	312	311	623				
2 children	15	12	27				
3 children	0	1	1				
No reported	2	6	8				
Total	329	330	659				
Gender of child 1				1.729	2	0.421	0.051
Female	102	115	217				
Male	224	210	434				
No reported	2	6	8				
Total	329	330	659				
Gender of child 2				0.105	2	0.949	0.013
Female	20	20	40				
Male	23	21	44				
N/A	284	283	567				
No reported	2	6	8				
Total	329	330	659				
Gender of child 3				0.144	2	0.931	0.015
Female	7	8	15				
Male	6	7	13				
N/A	314	309	623				
No reported	2	6	8				
Total	329	330	659				
Independence of child 1				1.426	4	0.840	0.047
No need for supervision or assistance	44	44	88				
Requires only supervision	139	142	281				
Needs assistance	75	79	154				
Needs assistance all the time	53	42	95				
N/A	16	17	33				
No reported	2	6	8				
Total	329	330	659				
Independence of child 2				1.756	4	0.780	0.052
No need for supervision or assistance	12	7	19				
Requires only supervision	10	9	19				
Needs assistance	5	5	10				
Needs assistance all the time	4	6	10				
N/A	296	297	593				
No reported	2	6	8				
Total	329	330	659				
Independence of child 3				1.534	4	0.821	0.049
No need for supervision or assistance	2	3	5				
Requires only supervision	3	1	4				
Needs assistance	1	1	2				
Needs assistance all the time	1	2	3				
N/A	320	317	637				
No reported	2	6	8				
Total	329	330	659				
Age of child 1 (years)				1.453	4	0.835	0.047
0–1	21	20	41				
1–3	122	133	255				
3–5	143	132	275				
6–18	35	31	66				
N/A	6	8	14				
No reported	2	6	8				
Total	329	330	659				
Age of child 2 (years)				0.643	4	0.958	0.031
0–1	3	2	5				
1–3	13	11	24				
3–5	8	7	15				
6–18	8	10	18				
N/A	295	294	589				
No reported	2	6	8				
Total	329	330	659				
Age of child 3 (years)				1.026	3	0.795	0.040
1–3	3	3	6				
3–5	1	3	4				
6–18	3	3	6				
N/A	320	315	635				
No reported	2	6	8				
Total	329	330	659				

**Table 3:** Observed frequency of variables related to the early intervention services received by families.

Variables	Sample 1	Sample 2	Total	*Χ* ^2^	*df*	*p*	Cramer’s *V*
Time receiving services				1.817	4	0.769	0.053
<6 months	96	92	188				
Between 6 and 12 months	80	70	150				
Between 12 and 23 months	97	101	198				
Between 24 and 48 months	42	50	92				
>48 months	14	17	31				
Total	329	330	659				
Frequency of the sessions/visits				7.731	9	0.561	0.108
None	3	1	4				
Every day	5	1	6				
Once a week	171	178	349				
Twice a week	74	84	158				
3 to 4 times a week	12	12	24				
Once every 2 weeks	37	27	64				
Once a month	19	17	36				
Once every 2 or 3 months	6	8	14				
Twice a year	1	0	1				
Once a year	1	2	3				
Total	329	330	659				
% of time you interact with the professional during sessions				2.671	5	0.750	0.064
None	12	11	23				
<25% of the session	36	32	68				
Between 26 and 50% of the session	18	19	37				
Between 51 and 95% of the session	34	27	61				
>95% of the session	219	235	454				
My child receives services at school, I am not present	10	6	16				
Total	329	330	659				

**Table 4:** Sample 1 factor loading weights of MPOC-20 items.

	Factor 1	Factor 2	Uniqueness
i1	**0.516**	0.107	0.637
i2	0.135	**0.626**	0.460
i3	**0.983**	−0.123	0.205
i4	**0.430**	0.438	0.332
i5	**0.786**	−0.016	0.402
i6	**0.529**	0.257	0.443
i7	0.444	**0.443**	0.303
i8	**0.540**	0.352	0.291
i9	**0.871**	0.016	0.219
i10	**0.895**	−0.025	0.234
i11	**0.505**	0.201	0.548
i12	**0.670**	0.196	0.310
i13	**0.900**	−0.142	0.368
i14	0.079	**0.700**	0.418
i15	0.302	**0.517**	0.400
i16	−0.007	**0.795**	0.377
i17	0.079	**0.753**	0.335
i18	0.150	**0.728**	0.278
i19	−0.114	**0.920**	0.303
i20	−0.180	**0.915**	0.384

*N = 329.* Factor 1 = *providing comprehensive care* and Factor 2 = *providing information.* Bolded-factor loadings represent the items assigned to each factor.

**Table 5:** MPOC-20 item factor loadings.

					95% CI		
Indicator	*λ*	*SE*	*Z*	*p*	*LL*	*UL*	*β*
Factor 1							
msf1	0.790	0.033	23.813	<0.001	0.725	0.855	0.737
msf3	0.585	0.026	22.692	<0.001	0.534	0.635	0.677
msf4	1.017	0.042	24.344	<0.001	0.935	1.099	0.820
msf5	0.783	0.033	24.034	<0.001	0.719	0.847	0.788
msf6	0.760	0.034	22.142	<0.001	0.693	0.828	0.701
msf8	0.910	0.036	25.573	<0.001	0.841	0.980	0.830
msf9	0.678	0.026	25.963	<0.001	0.627	0.730	0.768
msf10	0.728	0.030	23.944	<0.001	0.669	0.788	0.819
msf11	0.764	0.033	22.908	<0.001	0.698	0.829	0.670
msf12	0.806	0.031	25.829	<0.001	0.745	0.867	0.836
msf13	0.658	0.030	22.190	<0.001	0.600	0.716	0.747
Factor 2							
msf2	1.169	0.047	24.931	<0.001	1.077	1.261	0.760
msf7	1.067	0.043	24.714	<0.001	0.983	1.152	0.818
msf14	1.232	0.049	24.961	<0.001	1.135	1.329	0.779
msf15	1.004	0.041	24.421	<0.001	0.924	1.085	0.765
msf16	1.029	0.039	26.102	<0.001	0.951	1.106	0.716
msf17	1.239	0.046	26.992	<0.001	1.149	1.329	0.877
msf18	1.073	0.042	25.337	<0.001	0.990	1.156	0.797
msf19	1.337	0.049	27.017	<0.001	1.240	1.434	0.774
msf20	1.210	0.048	25.149	<0.001	1.115	1.304	0.661

*N = 330*. Factor 1 = *providing comprehensive care* and Factor 2 = *providing information*.

**Table 6:** McDonald’s ω and Cronbach’s α indexes by MPOC-20 factors for Samples 1 and 2.

	McDonald’s ω			Cronbach’s α		
	ω	95% CI LL	95% CI UL	α	95% CI LL	95% CI UL
Sample 1 (*N* = 329)						
Factor 1	0.943	0.927	0.955	0.941	0.932	0.949
Factor 2	0.934	0.920	0.945	0.932	0.922	0.941
Sample 2 (*N* = 330)						
Factor 1	0.935	0.916	0.949	0.934	0.924	0.943
Factor 2	0.924	0.906	0.939	0.922	0.910	0.933

Factor 1 = *providing comprehensive and supportive care* and Factor 2 = *providing information*.

## Data Availability

The data presented in this study are available on request from the corresponding author due to privacy and ethical restrictions.
